# Abdominal simultaneous 3D water T_1_ and T_2_ mapping using a free‐breathing Cartesian acquisition with spiral profile ordering

**DOI:** 10.1002/mrm.70040

**Published:** 2025-09-05

**Authors:** Jonathan Stelter, Kilian Weiss, Lisa Steinhelfer, Jakob Meineke, Weitong Zhang, Bernhard Kainz, Rickmer F. Braren, Dimitrios C. Karampinos

**Affiliations:** ^1^ Institute for Diagnostic and Interventional Radiology, School of Medicine and Health, TUM University Hospital Technical University of Munich Munich Germany; ^2^ Philips GmbH Market DACH Hamburg Germany; ^3^ Philips Innovative Technologies Hamburg Germany; ^4^ Department of Computing Imperial College London London UK; ^5^ Department Artificial Intelligence in Biomedical Engineering Friedrich‐Alexander‐Universität Erlangen‐Nürnberg (FAU) Erlangen Germany; ^6^ Laboratory of Magnetic Resonance Imaging Systems and Methods École Polytechnique Fédérale de Lausanne (EPFL) Lausanne Switzerland; ^7^ CIBM Center for Biomedical Imaging (CIBM) Lausanne Switzerland

**Keywords:** liver, relaxometry, Look‐Locker, isotropic resolution, water‐fat separation

## Abstract

**Purpose:**

To develop a method for abdominal simultaneous 3D water $T_{1}$ ($wT_{1}$) and $T_{2}$ ($wT_{2}$) mapping with isotropic resolution using a free‐breathing Cartesian acquisition with spiral profile ordering (CASPR) at 3 T.

**Methods:**

The proposed data acquisition combines a Look‐Locker scheme with the modified BIR‐4 adiabatic preparation pulse for simultaneous $wT_{1}$ and $wT_{2}$ mapping. CASPR is employed for efficient and flexible k‐space sampling at isotropic resolution during free breathing. The imaging pipeline includes subspace reconstruction, water‐fat separation, and $B_{0}$‐specific dictionary matching. The proposed method was validated in a water‐fat relaxometry phantom using spin echo‐based reference techniques and was compared with MOLLI $T_{1}$ and GRASE $T_{2}$ mapping in 10 volunteers. The method's flexibility was assessed at isotropic resolutions of 2.5, 3, and 3.5 mm, with corresponding scan times of 7:19, 5:23, and 3:52 min. Additionally, the method was applied to 9 oncological patients with abdominal pathologies.

**Results:**

Phantom experiments demonstrated good agreement between the proposed method and spin echo‐based reference techniques across a wide range of $wT_{1}$ and $wT_{2}$ values. The volunteer study assessed in vivo quantification performance and demonstrated the flexibility of the proposed method at different spatial resolutions. The 3 mm method was successively performed in patients with abdominal pathologies, whereby lesions with diameters below 1 cm could be assessed.

**Conclusion:**

The proposed Look‐Locker‐based method using CASPR enables efficient, flexible, and simultaneous $wT_{1}$ and $wT_{2}$ mapping at isotropic resolution in a fixed scan time during free breathing.

## 1 INTRODUCTION

Quantitative magnetic resonance imaging (MRI) is of increasing interest for abdominal imaging, particularly for the noninvasive assessment of chronic liver disease.^1^, ^2^, ^3^, ^4^ Water‐specific $T_{1}$ ($wT_{1}$) mapping has been proposed as a technique for evaluating liver fibrosis and inflammation,^5^, ^6^ correcting for the confounding effect of hepatic fat^7^, ^8^, ^9^ and without requiring additional hardware in contrast to MR elastography.^10^ The combination of $wT_{1}$ and $wT_{2}$ mapping can provide a more comprehensive tissue characterization in liver fibrosis,^10^, ^11^, ^12^, ^13^, ^14^ focal liver lesions,^15^, ^16^, ^17^ and in the assessment of $T_{2}$‐based liver iron concentration.^18^, ^19^ Furthermore, volumetric relaxometry methods enable whole‐organ assessment, which is particularly relevant for diseases with heterogeneous and mosaic‐like manifestations, such as primary or secondary sclerosing cholangitis.^20^, ^21^ Extending to multiorgan relaxometry can facilitate the evaluation of systemic diseases. Simultaneous assessment of abdominal organs may enhance the characterization of hepatic congestion,^22^ granulomatous diseases such as sarcoidosis,^23^, ^24^ inflammatory bowel disease,^25^ and facilitate lymph node mapping in conditions including lymphoma and metastatic disease^26^.

Several breath‐hold‐based $wT_{1}$ mapping techniques have been developed, with variable flip angle (VFA) methods allowing 3D liver coverage within the limited scan time.^27^, ^28^, ^29^ However, VFA methods are susceptible to transmit $B_{1}$ inhomogeneities and typically require $B_{1}$ mapping for correction. In contrast to breath‐hold‐based methods, free‐breathing acquisitions allow retrospective motion correction using suitable k‐space trajectories, improving patient comfort and typically providing greater spatial coverage. Free‐breathing abdominal MRI has often employed radial stack‐of‐stars trajectories,^30^, ^31^, ^32^, ^33^, ^34^ while Cartesian acquisitions with spiral profile ordering (CASPR) offer an alternative, allowing flexible sampling schemes with preserved motion correction capabilities, reduced susceptibility to errors caused by gradient delays and eddy currents, and simplified reconstruction via fast Fourier transform (FFT).^35^, ^36^, ^37^, ^38^ Existing free‐breathing liver $wT_{1}$ methods commonly use a Look‐Locker scheme with multiple $T_{1}$ contrasts acquired after each inversion pulse.^31^, ^32^, ^34^ Simultaneous $wT_{1}$ and $wT_{2}$ mapping has been achieved using a Look‐Locker scheme interleaved with T2prep pulses at 0.55 T^37^ and a modified inversion recovery approach acquiring a single contrast after each inversion or T2prep pulse at 3 T.^33^ Both methods acquire four volumes with distinct contrast weightings similar to a previously proposed framework for joint 3D $wT_{1}$ and $wT_{2}$ mapping at isotropic resolution in cardiac imaging.^39^ Additionally, MR multitasking (MT) has demonstrated simultaneous $T_{1}$ and $T_{2}$ mapping for cardiovascular imaging using a Look‐Locker scheme with an adiabatic preparation pulse that encodes $T_{1}$ and $T_{2}$,^40^ but limited to a 2D acquisition and without water‐fat separation.

Simultaneous $wT_{1}$ and $wT_{2}$ mapping can provide co‐registered parameter maps, increase the acquisition efficiency, and mitigate biases in the respective parameter estimates, especially for $T_{1}$ as a confounding factor in T2prep‐ and gradient‐echo‐based $T_{2}$ mapping.^41^, ^42^, ^43^ Despite potential benefits of simultaneous 3D $wT_{1}$ and $wT_{2}$ mapping in the abdomen, technical implementations are limited. Look‐Locker‐based methods are efficient for $T_{1}$ mapping, but integrating $T_{2}$ mapping poses challenges due to prolonged scan times. In addition, due to long shot durations, it is usually not possible to achieve large spatial coverage for multi‐organ assessment, or to achieve isotropic resolution with a Look‐Locker acquisition in combination with a radial stack‐of‐stars trajectory.

In this work, a novel approach for simultaneous 3D $wT_{1}$ and $wT_{2}$ mapping is proposed using a Look‐Locker scheme with a CASPR trajectory. CASPR enables large field‐of‐view (FOV) abdominal imaging at isotropic resolution and flexibility in the selection of the spatial resolution with fixed sequence timings. To ensure clinically feasible scan times and high accuracy while accounting for confounding factors, a subspace reconstruction approach is employed that incorporates a low‐rank constraint, corrects for $T_{1}$ relaxation blurring, and is combined with water‐fat separation and $B_{0}$‐specific dictionary matching.

## 2 METHODS

### 2.1 Pulse sequence

**FIGURE 1:**
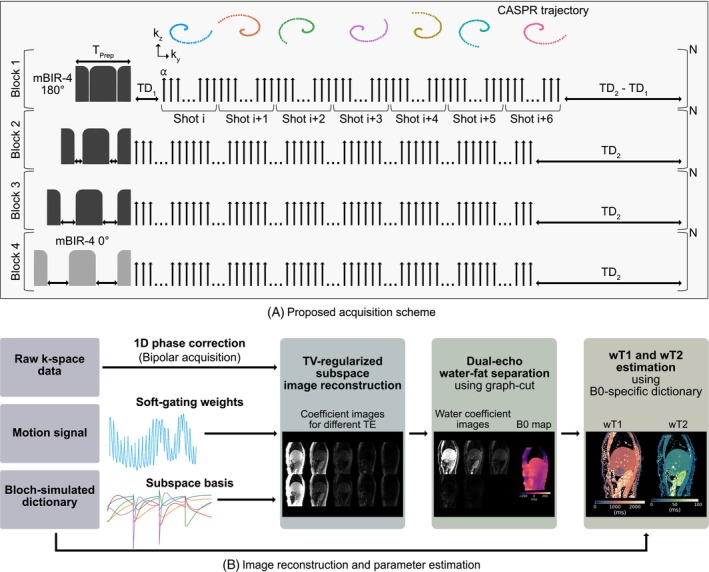
Schematic overview of (A) the proposed acquisition scheme and (B) the image reconstruction and parameter estimation pipeline. (A) The acquisition consists of four blocks, each containing an adiabatic preparation pulse followed by a Look‐Locker readout. A golden‐step CASPR trajectory was applied varying every shot the sampled profiles. (B) The undersampled raw k‐space data were reconstructed using a soft‐gated, total‐variation (TV)‐regularized subspace reconstruction. The subspace reconstruction was followed by water‐fat separation and $wT_{1}$ and $wT_{2}$ estimation using a $B_{0}$‐specific dictionary approach.

The proposed pulse sequence was developed to encode $T_{1}$ and $T_{2}$ simultaneously using an adiabatic preparation pulse and a Look‐Locker gradient echo readout (Figure 1A).

The preparation pulse used is the modified B1‐insensitive rotation pulse (mBIR)‐4^43^, ^44^, ^45^ with frequency sweep = 3700 Hz, amplitude = 13.5μT and duration without gap = 10 ms. The sequence consists of four different 3 s blocks, each repeated N times. N denotes the number of repetitions for each block and depends on the acceleration factor and the acquisition matrix size. Each block consists of the preparation pulse, the Look‐Locker gradient echo readout and a pause ${\text{TD}}_{2}$. The gaps of the preparation pulse are varied in each block to achieve different $T_{2}$ weightings (total pulse durations $T_{\text{Prep}}$ = [10.9, 25.9, 40.9, 55.9] ms). The preparation pulse is generally used as an inversion pulse (180° pulse angle), with the exception of the last block, in which a T2prep pulse with 0° pulse angle is used to increase the $T_{2}$ encoding capability of the acquisition scheme. In the first block, an additional delay ${\text{TD}}_{1}=200$ ms is employed between the preparation pulse and the Look‐Locker readout.

In each block, the Look‐Locker gradient echo readout consists of seven shots with 60 acquired profiles per shot. Two echoes are acquired in one $T_{R}$ using alternating readout gradients. A CASPR trajectory^46^ is employed to acquire profiles at different $k_{y}$ and $k_{z}$ locations in an outward spiral starting with the k‐space center in each shot. The spiral profile ordering rotates for each shot with golden angle increment and uses an acceleration factor of R=7.5 for each of the 4×7 contrasts. If the acquisition matrix size changes, the total scan time changes, while the other sequence timings remain fixed.

### 2.2 Image reconstruction and parameter estimation

The image reconstruction and parameter estimation pipeline are depicted in Figure 1. Preprocessing was performed to correct for echo misalignment, compute weights for motion soft‐gating, and estimate a subspace basis. Subsequently, TV‐regularized subspace reconstruction, a dual‐echo‐based water‐fat separation, and $B_{0}$‐specific dictionary matching for $wT_{1}$ and $wT_{2}$ estimation was performed.

#### 2.2.1 Echo misalignment correction

Raw k‐space data were corrected in the frequency‐encoding direction for echo misalignment due to the bipolar data acquisition.^47^ The constant shift in k‐space was estimated using calibration lines in frequency‐encoding direction (at $k_{y}=k_{z}=0$) acquired with opposite gradient polarities before the actual scan. The shift was estimated and corrected by fitting and applying a linear phase in image space after 1D FFT.^48^

#### 2.2.2 Soft‐gating weights

The signal from an external motion sensor m[n] was extracted to compute soft‐gating weights $w_{\text{motion}}[n]$ corresponding to each k‐space profile n. The weights were used to adjust the influence of each k‐space profile in the reconstruction based on their similarity to a predefined reference state, the end‐expiration state, $m_{\text{ref}}$.^49^, ^50^ Similarity was assessed based on the difference in relative motion amplitude. The soft‐gating weights were derived using a Gaussian function:

(1) $$w_{\text{motion}}[n]=\exp\left(-\frac{{\left(m[n]-m_{\text{ref}}\right)}^{2}}{2\sigma^{2}}\right),$$

where the variance was defined based on the distance between the reference state and the second closest state $m_{\text{ref+2}}$, ensuring that the full width at half maximum (FWHM) of the Gaussian corresponds to this distance:

(2) $$\sigma =\frac{\left|m_{\text{ref+2}}-m_{\text{ref}}\right|}{2\sqrt{2\ln2}}.$$

Motion states were defined to estimate the reference state and the variance for soft‐gating. Specifically, four motion states were defined using k‐medoids clustering.

#### 2.2.3 Subspace estimation and reconstruction

Image reconstruction was performed in a joint $T_{1}$ and $T_{2}$ relaxation subspace for each echo separately. A basis $\Phi_{k}$ of this linear subspace was computed by singular value decomposition (SVD)^51^ from a Bloch‐simulated dictionary D containing the profile‐resolved temporal signals (4 blocks × 7 shots × 60 profiles per shot). The Bloch simulation modeled the RF amplitude and frequency modulation of the mBIR‐4 pulse and the gradient echo readout without considering $T_{2}^{*}$ decay for the two different echo times. The fat fraction was not modeled in the dictionary because water and fat proton densities are linear parameters in the gradient echo signal and their combined signal lies also in the same subspace.^52^ The resulting dictionary contained the transverse magnetization during each k‐space profile acquisition as entry for different parameters: $B_{0}\in \{-200,-180,\ldots ,200\}\;\text{Hz}$, $T_{1}\in \{105,120,\ldots ,1500\}\cup \{1550,1600,\ldots ,3000\}\;\text{ms}$, $T_{2}\in \{9,10.5,\ldots ,120\}\;\text{ms}$.

The first k=5 right singular vectors $\varphi_{i}$ were chosen to form the basis of the linear subspace $\Phi_{k}=\{\varphi_{1},..,\varphi_{k}\}$ representing 97% of the signal variability in the dictionary.

The k coefficient images x at the different echo times were reconstructed from the undersampled k‐space data y by solving the following inverse problem:

(3) $$x=\underset{x^{\prime }}{arg min}{\left\|𝒲𝒫ℱ𝒮x^{\prime }-y\right\|}_{2}^{2}+\lambda \overset{k}{\underset{i=1}{\sum }}\alpha_{i}{\left\|\text{TV}(x_{i}^{\prime })\right\|}_{1},$$

with 𝒲 is a weighting operator containing the sampling density compensation multiplied by the respiratory soft‐gating weights $w_{\text{motion}}[n]$, 𝒫 maps the temporal subspace $\Phi_{k}$ to the space of acquired profiles, ℱ represents the 3D Fourier transform, and 𝒮 denotes the coil sensitivity maps estimated from a pre‐scan. The 𝒫 operator commutes with the ℱ and 𝒮 operators.^52^ Spatial total variation (TV) regularization was applied to exploit spatial sparsity based on a fast gradient projection algorithm.^53^ The TV regularization term for each coefficient image $x_{i}$ was scaled by the normalized singular value $\alpha_{i}$ of the corresponding basis vector to compensate for intensity differences across the coefficient images. The alternating direction method of multipliers (ADMM) was applied as solver for the reconstruction problem.

#### 2.2.4 $B_{0}$‐specific dictionary matching

A field map for the first coefficient image was estimated using a dual‐echo graph‐cut algorithm,^33^, ^54^ incorporating a water‐fat signal model that accounts for the multi‐peak fat spectrum.^55^ The estimated field map was then employed to estimate water and fat images for each of the k coefficient images.

Dictionary matching took into consideration $B_{0}$ effects during the magnetization evolution, to especially account for the sensitivity of the mBIR‐4 pulse to $B_{0}$ variations. Specifically, dictionary matching was performed using the k water coefficient images and the simulated dictionary D with similar $T_{1}$ and $T_{2}$ range, but extended $B_{0}$ range to avoid dictionary truncation artifacts in regions with large $B_{0}$ inhomogeneities: $B_{0}\in \{-300,-280,\ldots ,300\}\;\text{Hz}$. The dictionary was projected into the subspace using the basis $\Phi_{k}$.^51^ For each voxel, the $B_{0}$‐specific subdictionary was selected based on the field map estimate, and the water coefficient estimates were matched to the sub‐dictionary by minimizing the $L_{2}$ difference between the simulated and measured signals.^33^

### 2.3 Experiments

The performance of the proposed method was evaluated in simulation, phantom, volunteer, and patient studies. The accuracy was assessed with regard to reference methods and the reproducibility was assessed using acquisitions at different spatial resolutions.

Bloch simulations were performed to evaluate the $B_{1}$ sensitivity of the proposed method for representative abdominal relaxation parameters ($T_{1}$ = 500:50:1500 ms, $T_{2}$ = 15:2.5:65 ms) and for $B_{1}$ = 0.6:0.1:1.2, reflecting typical $B_{1}$ inhomogeneities observed in the liver at 3 T.^56^ The simulated signals were projected onto the subspace, matched to the pre‐computed dictionary with $B_{1}=1$, and the estimation error was computed as the difference between the estimated and true relaxation times.

The phantom study employed a commercial water‐fat $T_{1}$ phantom (Calimetrix, Madison, WI, USA) with varying $wT_{1}$ and fat fraction (up to around 12%), in addition to 5 custom‐built phantom vials to increase the range for $wT_{2}$ in the phantom. The custom‐built phantom contains different concentrations of manganese(II) chloride, with one vial having a fat content of about 25%, while the other vials contain no fat. A 6‐echo gradient echo Dixon scan scan ($T_{E,1}=1.35$ ms, $\Delta T_{E}=1.05$ ms, $T_{R}=7.8$ ms, flip angle = 3°) was used to estimate the fat fraction.

The volunteer study comprised 10 subjects, while the patient study included 9 subjects with different abdominal diseases including primarily patients with HCC. The study was approved by local institutional review board (Klinikum rechts der Isar, Technical University of Munich, Munich, Germany) and informed consent was given by volunteers and patients.

#### 2.3.1 Measurements

The proposed method was acquired at three different spatial resolutions in phantom and volunteer studies (2.5, 3 and 3.5 mm isotropic resolution). The different matrix sizes were acquired with a similar pulse sequence without adapting the duration of the shots or other timings allowing the use of the same pre‐computed dictionary across resolutions. Changing the matrix size only varied the sampled profiles in the $k_{y}-k_{z}$ grid and changed the amount of sampled profiles and scan time.

Proposed $wT_{1}$ maps were compared to vendor‐implemented MOLLI $T_{1}$ in phantom and volunteer studies and to Dixon inversion recovery spin echo (IR‐SE) $wT_{1}$ in phantom studies. Proposed $wT_{2}$ maps were compared to vendor‐implemented GRASE $T_{2}$ in phantom and volunteer studies and to Dixon spin echo (SE) $wT_{2}$ in phantom studies. Dixon IR‐SE and Dixon SE sequences correct for fat‐confounding effects using two acquisitions with the second acquisition shifted with $\Delta T_{E}=1$ ms, but could not be employed in in vivo studies due to long acquisition times. The sequence parameters for the different methods are summarized in Table 1. The proposed method with 3 mm isotropic resolution was acquired in patients in addition to conventional sequences used at our institution. Sequence parameters for the clinical sequences are summarized in Supporting Information Table S1.

**TABLE 1:** Sequence parameters used in the phantom, volunteer, and patient study.

	Proposed method	MOLLI	GRASE	Dixon IR‐SE	Dixon SE
Parameter	$wT_{1}/wT_{2}$	$T_{1}$	$T_{2}$	$wT_{1}$	$wT_{2}$
Motion compensation	Free‐breathing	Breath‐hold	Respiratory trigger	Phantom‐only	Phantom‐only
Scan orientation	Sagittal	Axial	Axial	Axial	Axial
Voxel size (${\text{mm}}^{3}$)	2.5×2.5×2.5/3×3×3/3.5×3.5×3.5	2.5×2.5×5	3×3×3	2.5×2.5×8	
FOV (RL×AP×FH, ${\text{mm}}^{3}$)^a^	350×252.8×350 / 351×262.5×350 / 353.5×262.5×350	400×400×5	400×400×200	260×178×8	
$T_{E}$ (ms)	[1.0, 2.1]	1.1	[16, 24, 32, 40, 48, 56, 64, 72]	8^b^	[10,20,30,40,50]^b^
$T_{I}$ (ms)	[6, 210, 414, 618, 822, 1026, 1230]^c^	5(3)3 with min. $T_{I}$: 113.1/350	–	[150, 210, 300, 500, 1000, 1500, 2400]	–
$T_{R}$ (ms)	3.4	2.2	1546.8	8000	3000
Flip angle (°)	3	20	90	90	90
Scan‐specific timings (ms)	$T_{\text{Prep}}$ = [10.9, 25.9, 40.9, 55.9], ${\text{TD}}_{1}$ = 1554, ${\text{TD}}_{2}$ = 200	Variable heartbeat interval	–	–	–
Acceleration	R = 7.5, $R_{\mathrm{eff}}$ = (18 ± 7) after soft‐gating	CS‐SENSE (R = 3)	SENSE (R = 2)	–	–
Scan time (min:s)	7:19 / 5:23 / 3:52	0:11	2:24 (nominal)	134:24	36:00

*Note*: The proposed method was compared in the phantom with Dixon inversion recovery spin echo (IR‐SE) $wT_{1}$ and Dixon spin echo (SE) $wT_{2}$ mapping, in addition to the vendor‐implemented MOLLI $T_{1}$ and GRASE $T_{2}$ sequences. The fat‐corrected Dixon IR‐SE and Dixon SE techniques were not employed in vivo due to long acquisition times. The effective acceleration factor $R_{\text{eff}}$ after soft‐gating is computed by weighting each k‐space profile according to its corresponding soft‐gating weight, with a maximum value of 1 for oversampled profiles.

^a^ RL: right‐left, AP: anterior‐posterior, FH: feet‐head; FOV in AP direction was extended in some patients due to the body size.

^b^ Dixon multi‐acquisition with echo time shift $\Delta T_{E}=1\;\text{ms}$ for second acquisition.

^c^ Additional delay ${\text{TD}}_{1}=200$ ms for the first block, leading to $T_{I}=[206,410,614,818,1022,1226,1430]$ ms.

Measurements were performed on a clinical 3 T scanner (Ingenia Elition X, Philips Healthcare, The Netherlands) using a 16‐channel anterior coil and 12‐channel posterior coil for in vivo studies. Phantom measurements used a 16‐channel head coil. Motion signals for soft‐gating were acquired using a respiratory‐tracking camera provided by the vendor (VitalEye, Philips Healthcare, The Netherlands).^57^, ^58^ For one volunteer, a respiratory belt was used for motion tracking due to unavailability of the camera.

#### 2.3.2 Data processing

Image reconstruction was performed in Julia 1.9, and Bloch simulations, water‐fat separation and dictionary matching were performed in Python 3.10. Source code and phantom data will be made publicly available: https://github.com/BMRRgroup/abdominal‐t1t2‐mapping.

k‐space data were pre‐whitened using the noise covariance matrix estimated together with the coil sensitivity maps from a reference scan using proprietary software (ReconFrame, Gyrotools). Regularization parameter λ=0.001 was chosen in the solution of Eq. 3 to reduce undersampling artifacts while preserving image details. The ADMM solver was employed with penalty ρ=0.01 and a balanced scheme for varying ρ.^59^ Water‐fat separation employed a 9‐peak in vivo fat model for volunteer and patient studies,^60^, ^61^ whereas a 9‐peak peanut oil model with correction for temperature effects (shift of 0.14 ppm of the water resonance) was employed for the phantom study.^62^ The color‐map recommendation for MR relaxometry maps was used for visualization^63^ and parameter maps for the proposed method were masked based on 10% of the 95th percentile intensity of the water image.

MOLLI and GRASE parameter maps were reconstructed online, while Dixon IR‐SE and Dixon SE images were reconstructed online with offline fitting using a 3‐parameter inversion recovery model for $T_{1}$ mapping and a 2‐parameter mono‐exponential model for $T_{2}$ mapping.

#### 2.3.3 Evaluation

To assess bias and precision across different spatial resolutions, cubic regions of interest (ROIs) of size 12mm×12mm×12mm were placed in the phantom $wT_{1}$ and $wT_{2}$ maps acquired with the proposed method. For the comparison methods, 2D ROIs of size 12mm×12mm were used.

In the volunteer study, 2D ROIs (12mm×12mm) were placed in two regions of the liver and in the back muscle to evaluate the accuracy in comparison with MOLLI and GRASE. Additional abdominal organs were not included in this comparison, as MOLLI is limited to single‐slice acquisition. For the reproducibility analysis across the three spatial resolutions, ROIs were placed in addition in the pancreas and spleen for 5 representative volunteers.

The accuracy compared to the reference methods and the reproducibility across the different resolutions were evaluated using linear correlation and Bland‐Altman analysis. The coefficient of variation (CV) was evaluated to assess the precision of the proposed method across the three different spatial resolutions in different ROIs.^64^

A qualitative assessment by a radiologist was performed to score the overall image quality and image sharpness of the $wT_{1}$ and $wT_{2}$ maps as well as the PD‐like water image in patients using a 5‐point Likert scale (1: non‐diagnostic, 2: poor, 3: moderate, 4: good, 5: excellent). The overall image quality was evaluated based on the impact of motion artifacts, susceptibility effects, $B_{1}$ inhomogeneity, and other artifacts. The image sharpness was evaluated based on the perceived versus nominal spatial resolution. Images from the clinical exam were examined as a reference.

## 3 RESULTS

Supplementary Figure S1 shows simulated $T_{1}$ and $T_{2}$ estimation errors as a function of the $B_{1}$ inhomogeneity. The simulation results demonstrate small $T_{1}$ and $T_{2}$ errors for relaxation times characteristic of healthy liver tissue, while the sensitivity to $B_{1}$ increases for longer $T_{1}$ and $T_{2}$ values.

### 3.1 Phantom study

**FIGURE 2:**
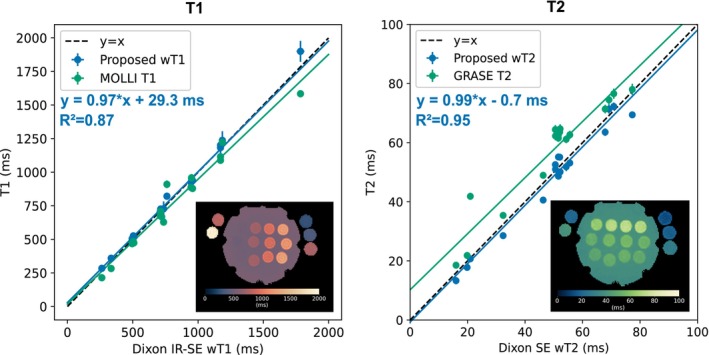
Validation of the proposed method at 3 mm isotropic resolution in a water‐fat relaxometry phantom. The $wT_{1}$ and $wT_{2}$ estimates of the proposed method show excellent agreement with Dixon inversion‐recovery spin‐echo (Dixon IR‐SE) $wT_{1}$ and Dixon spin‐echo (Dixon SE) $wT_{2}$ mapping. In addition, the performance of the proposed method is compared to vendor‐implemented MOLLI $T_{1}$ and GRASE $T_{2}$ mapping techniques.

Figure 2 shows results in the phantom that demonstrate good agreement of the proposed 3 mm method with the Dixon IR‐SE and Dixon SE references for a large $wT_{1}$ and $wT_{2}$ range. The largest $wT_{1}$ deviation from the reference of around 115 ms is observed for the vial with the largest $wT_{1}$ value of around 1800 ms. The mean differences between the proposed method and the references were (12±22) ms for $wT_{1}$ and (−1.4±3.1) ms for $wT_{2}$. Supplementary Figure S2 compares the differences in measuring wT1 and wT2 of the proposed method, MOLLI and GRASE against the reference methods as a function of fat fraction. The comparison shows no noticeable dependence on the fat fraction for the proposed method, while MOLLI and GRASE tend to positively correlate with the fat fraction. In the phantom vials without fat, MOLLI underestimated $T_{1}$, while GRASE overestimated $T_{2}$.

Phantom reproducibility results at three different isotropic resolutions are presented in Supplementary Figure S3. Bland‐Altman analysis shows no systematic bias across resolutions, though variance slightly increases at lower resolution. The mean differences between the 3.5 and 3 mm scans were (0.4±19.9) ms for $wT_{1}$ and (0.3±0.8) ms for $wT_{2}$.

### 3.2 In vivo study

**FIGURE 3:**
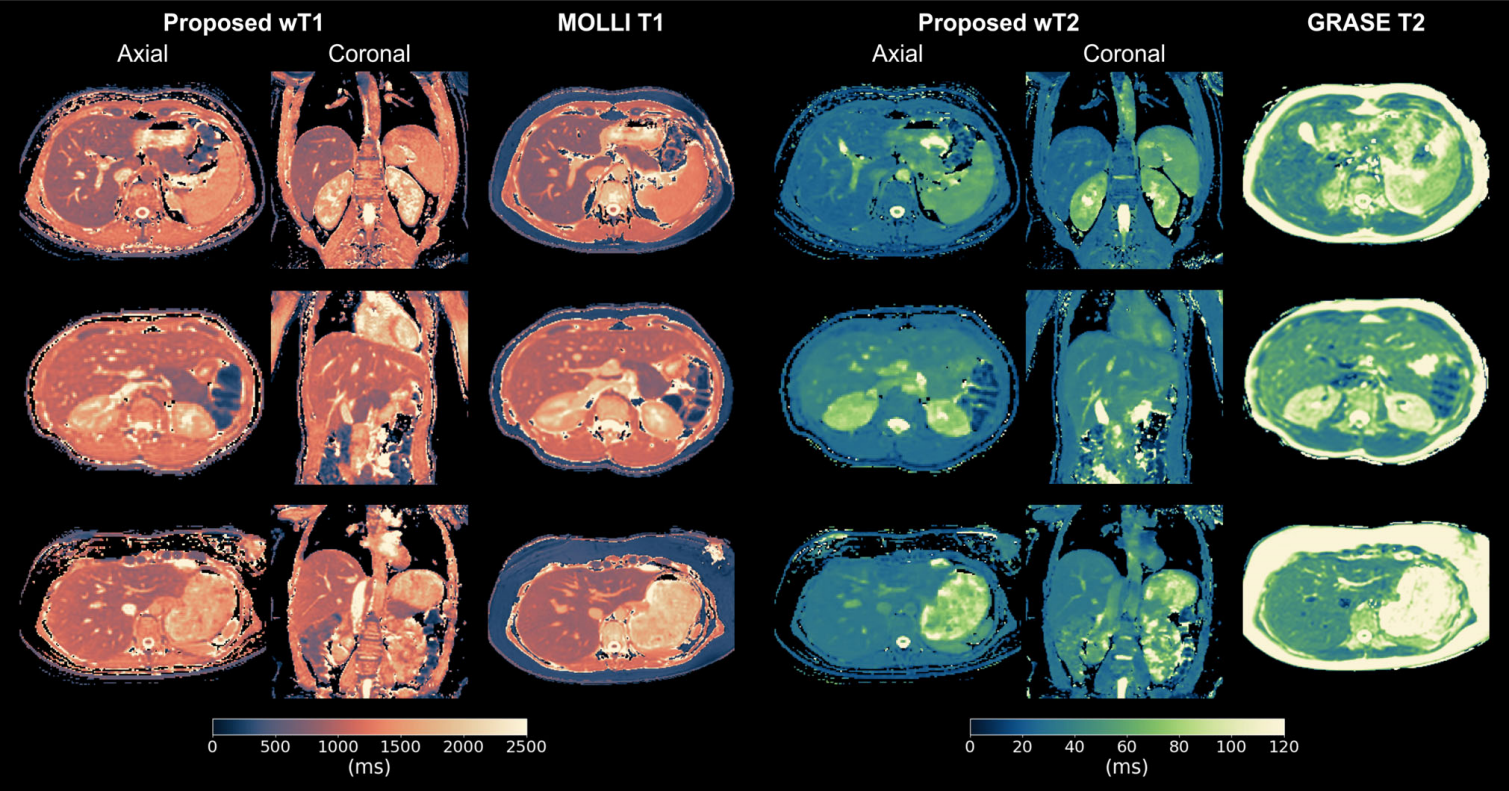
Visual comparison of $wT_{1}$ and $wT_{2}$ maps from the proposed 3D method (3 mm isotropic resolution) with MOLLI $T_{1}$ and GRASE $T_{2}$ in three volunteers. The proposed method is presented in both axial and coronal views, while the 2D MOLLI and GRASE maps are shown in the axial plane.

**FIGURE 4:**
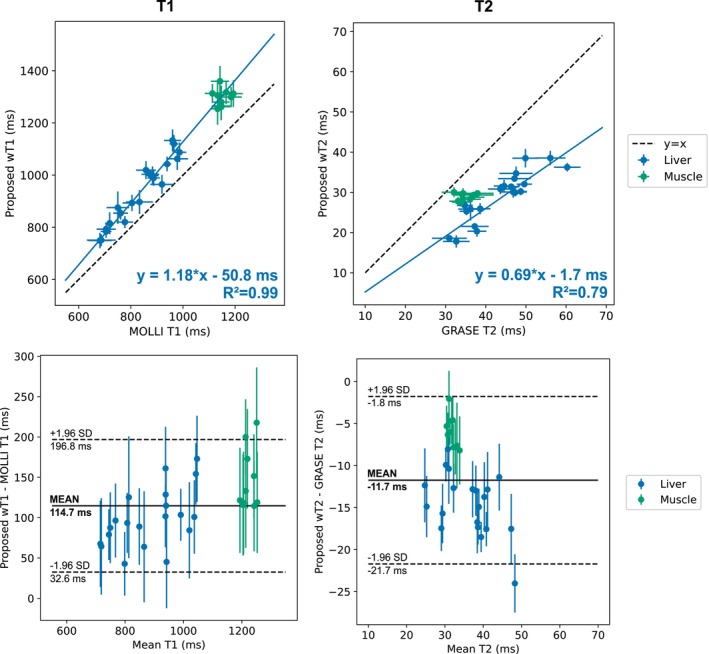
Quantitative comparison of the proposed method with MOLLI $T_{1}$ and GRASE $T_{2}$ mapping for the volunteer study. ROIs were placed in the liver and back muscle for all ten volunteers. Above is the linear correlation for $T_{1}$ and $T_{2}$. Below are Bland‐Altman plots comparing the differences between the methods. The lower $T_{1}$ values for MOLLI and the higher $T_{2}$ values for GRASE agree with the results in the phantom. The phantom references, Dixon IR‐SE, and Dixon SE, cannot be acquired in vivo due to the long acquisition times.

In vivo 3 mm $wT_{1}$ and $wT_{2}$ maps from three volunteers are presented in Figure 3, demonstrating the large FOV along the feet‐head (FH) direction. The in‐plane spatial resolution of the proposed $wT_{1}$ maps is comparable to breath‐hold‐based MOLLI $T_{1}$ at 2.5 mm in‐plane resolution. The proposed $wT_{2}$ maps exhibit slightly less distinct vessel delineation than proposed $wT_{1}$ but show improved quality compared to respiratory‐triggered GRASE $T_{2}$, which showed to be more prone to motion artifacts. The quantitative results in Figure 4 follow similar trends observed in the phantom experiments, with the proposed method generally yielding higher $T_{1}$ estimates than MOLLI (mean difference: (114±42) ms) and lower $T_{2}$ estimates than GRASE (mean difference: (−12±5) ms). Variability in $T_{1}$ and $T_{2}$ measurements across liver ROIs was substantially higher than in back muscle ROIs among volunteers.

Supplementary Videos S1 and S2 present animations for axial and coronal views of the proposed 2.5 mm $wT_{1}$ and $wT_{2}$ maps as well as the PD‐like water images. The reconstruction exhibits some residual stripe artifacts, primarily in the anterior‐posterior direction near the heart and in the lower abdomen. The artifacts are most pronounced in the PD‐like water images, while the $wT_{1}$ and $wT_{2}$ maps are less affected.

**FIGURE 5:**
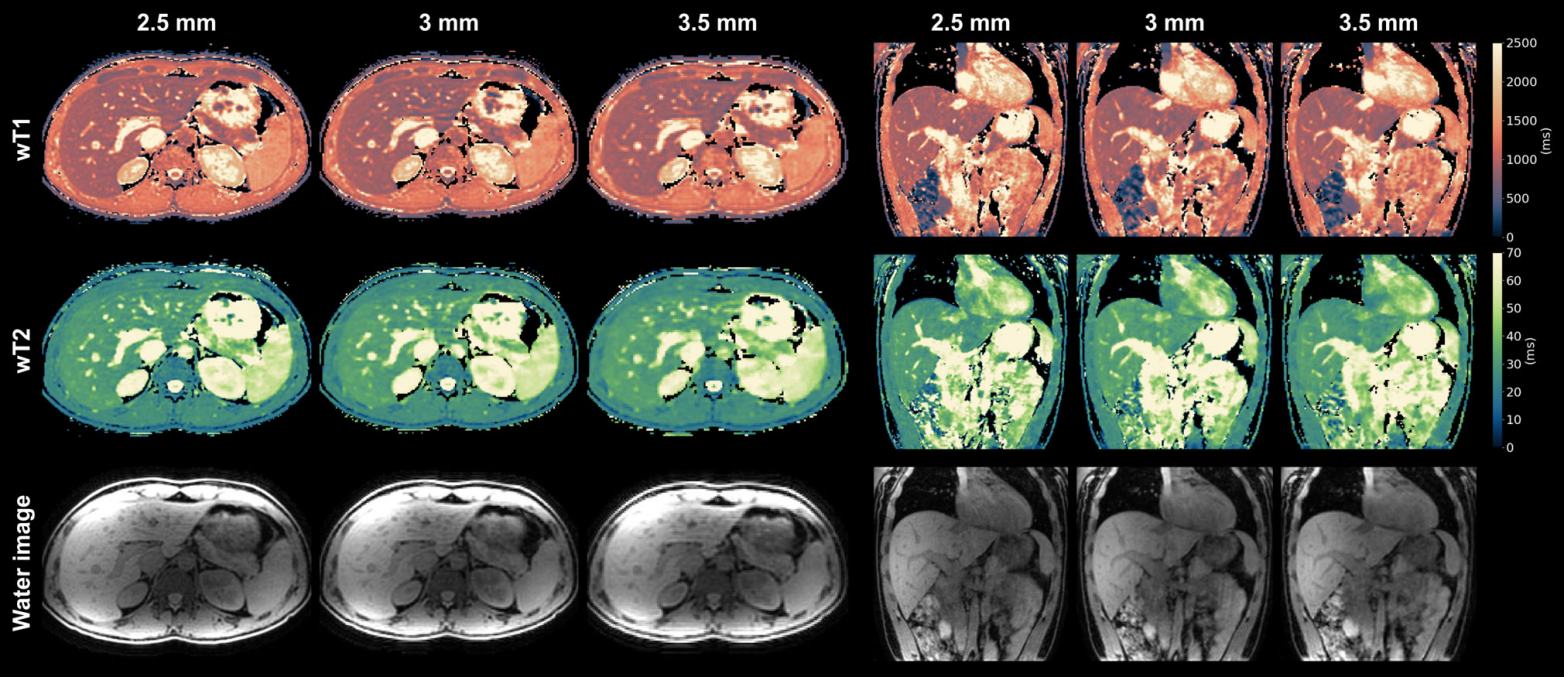
Visual comparison of the proposed method at 2.5, 3, and 3.5 mm isotropic resolution for the estimated $wT_{1}$ and $wT_{2}$ maps, as well as PD‐like water images. Axial (left) and coronal (right) views are shown for a representative volunteer.

**FIGURE 6:**
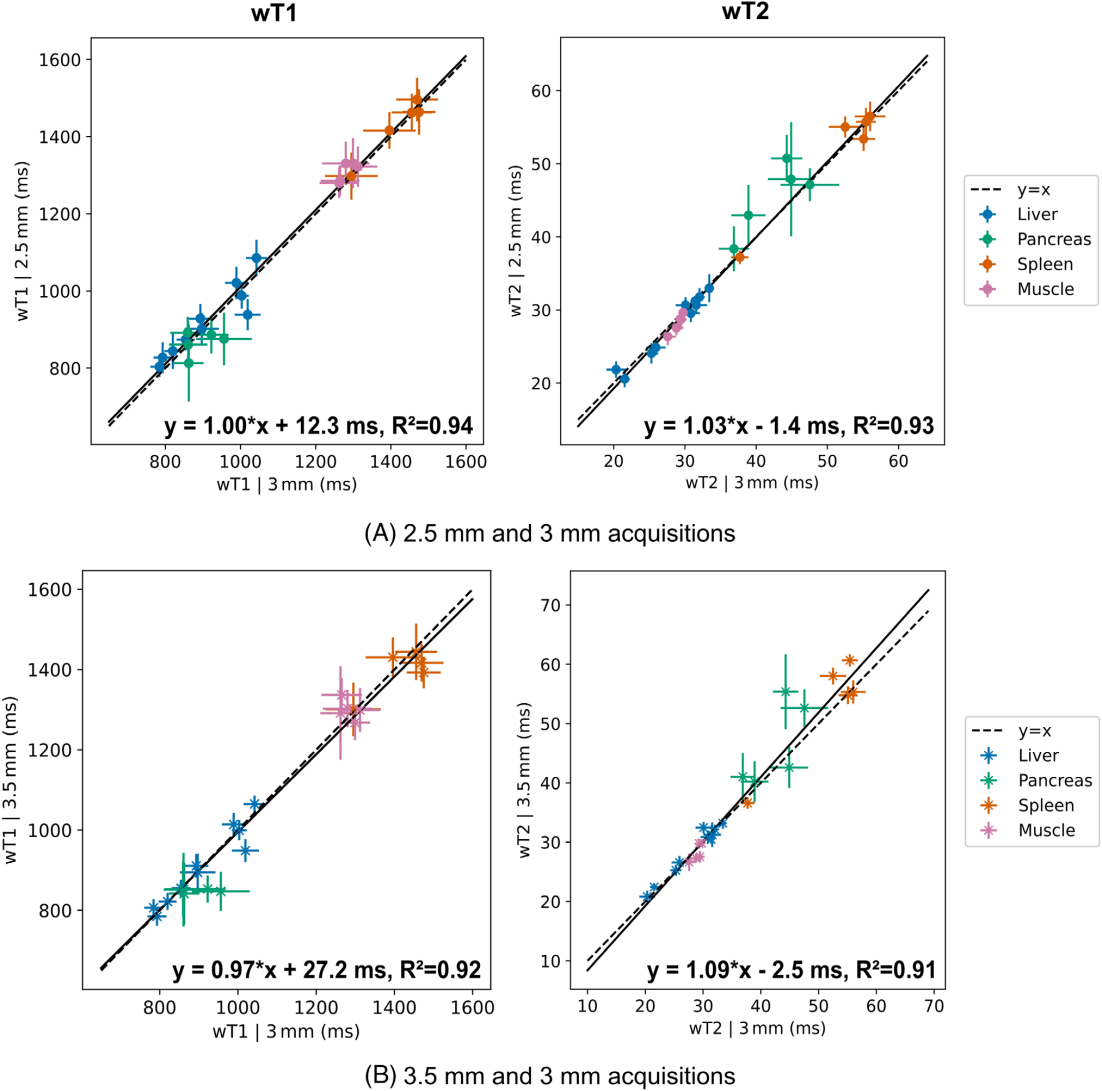
Reproducibility analysis of the proposed method at different spatial resolutions without repositioning in five representative volunteers. ROIs in the liver, pancreas, spleen, and muscle are compared at (a) 2.5 mm and (b) 3.5 mm isotropic resolution against the 3 mm acquisition. The results show a strong correlation across all analyses, with the largest variations observed in pancreatic ROIs.

Figure 5 presents $wT_{1}$ and $wT_{2}$ maps, along with PD‐like water images, acquired using the proposed method at isotropic resolutions of 2.5, 3, and 3.5 mm for a representative volunteer. The higher apparent spatial resolution at 2.5 mm is evident, with improved visibility of blood vessels in the liver and enhanced depiction of the abdominal organs. Reproducibility results show strong agreement across all three resolutions based on the linear correlations in Figure 6, with a slightly increased variance in the comparison between 3.5 and 3 mm acquisitions, as shown by the Bland‐Altman analysis in Supplementary Figure S4. The largest variations in $wT_{1}$ and $wT_{2}$ estimates were observed in ROIs within the pancreas. Supplementary Figure S5 compares the CV in ROIs across the three resolutions. Supplementary Table S2 reports the mean and range of measured $wT_{1}$ and $wT_{2}$ in liver, pancreas, spleen and muscle.

**FIGURE 7:**
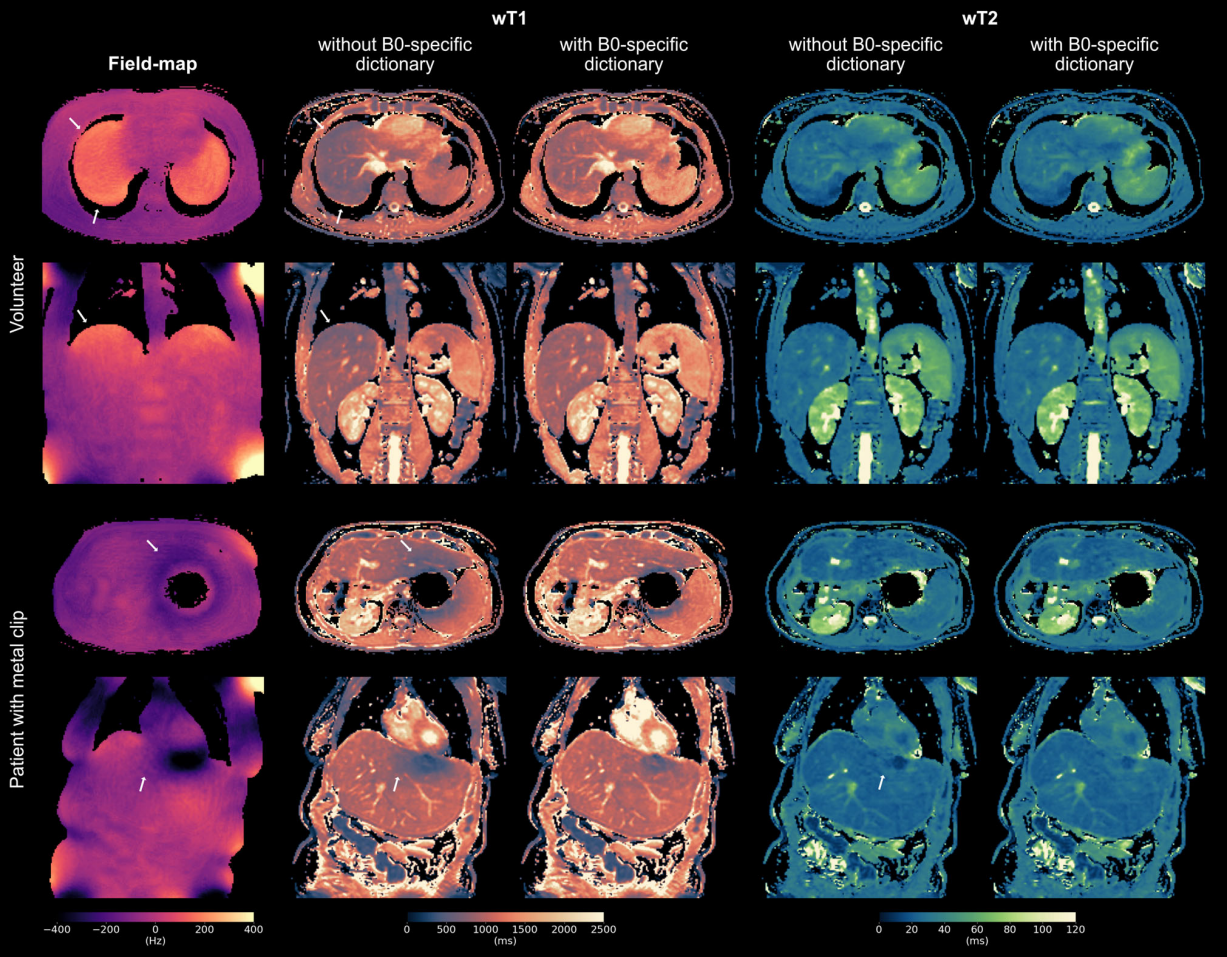
$B_{0}$ inhomogeneities can lead to quantification errors if they are not taken into account during dictionary matching. Proposed $wT_{1}$ and $wT_{2}$ maps are compared with and without considering $B_{0}$ in dictionary matching. The estimated field map from the data is shown as a reference. Strong $B_{0}$ inhomogeneities are observed in a volunteer in the upper liver region near the lung (top) and in a patient with a metal clip, leading to signal extinction (bottom). The regions are indicated by arrows highlighting primarily underestimated $wT_{1}$ without $B_{0}$‐specific dictionary matching.

Figure 7 shows results without and with the $B_{0}$‐specific dictionary matching approach in the presence of strong $B_{0}$ inhomogeneities for a volunteer and a patient. $B_{0}$ inhomogeneities of several hundred Hz primarily affected $wT_{1}$ maps when not accounted for in dictionary matching, whereas $wT_{2}$ remained more robust. The $B_{0}$‐specific approach mitigated $wT_{1}$ underestimation, as demonstrated in the upper liver region of a volunteer and in a patient with a metal clip, where $wT_{1}$ and $wT_{2}$ appear homogeneous in the liver even near a signal extinction region.

### 3.3 Patient cases

The overall image quality (4.4±0.7) and image sharpness (4.6±0.7) were both rated as good to excellent across the patient cohort on a 5‐point Likert scale (Supplementary Table S3). In one patient, the overall image quality was rated as moderate due to the presence of ascites and associated $B_{1}$ inhomogeneity artifacts.

**FIGURE 8:**
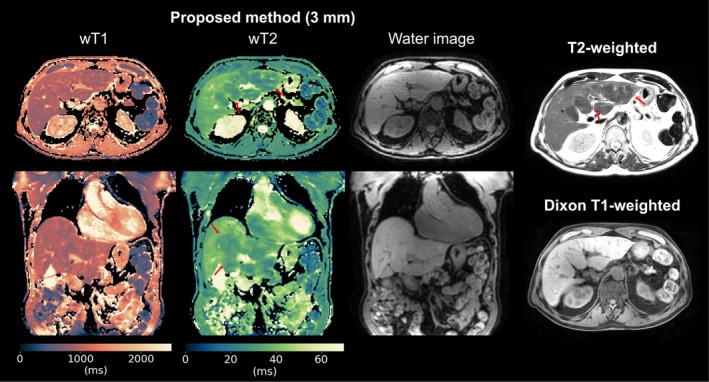
Proposed $wT_{1}$ and $wT_{2}$ mapping in a patient with ischemic cholangiopathy. 3 mm $wT_{1}$ and $wT_{2}$ maps, as well as PD‐like water images, are presented in axial and coronal views and compared to $T_{2}$‐weighted and native Dixon $T_{1}$‐weighted images from the clinical protocol. Red arrows indicate a liver region with lower $wT_{2}$, co‐localized with signal reduction on the clinical $T_{2}$‐weighted images.

Figure 8 presents results in a patient with ischemic cholangiopathy, which likely represents secondary sclerosing cholangitis and is characterized by multiple short‐segment strictures in the intrahepatic bile ducts following an intensive care unit stay. The proposed $wT_{2}$ map shows two liver regions with different $wT_{2}$ values but similar $wT_{1}$. The liver region with lower $wT_{2}$ is co‐localized with the signal reduction in the clinical $T_{2}$‐weighted image.

**FIGURE 9:**
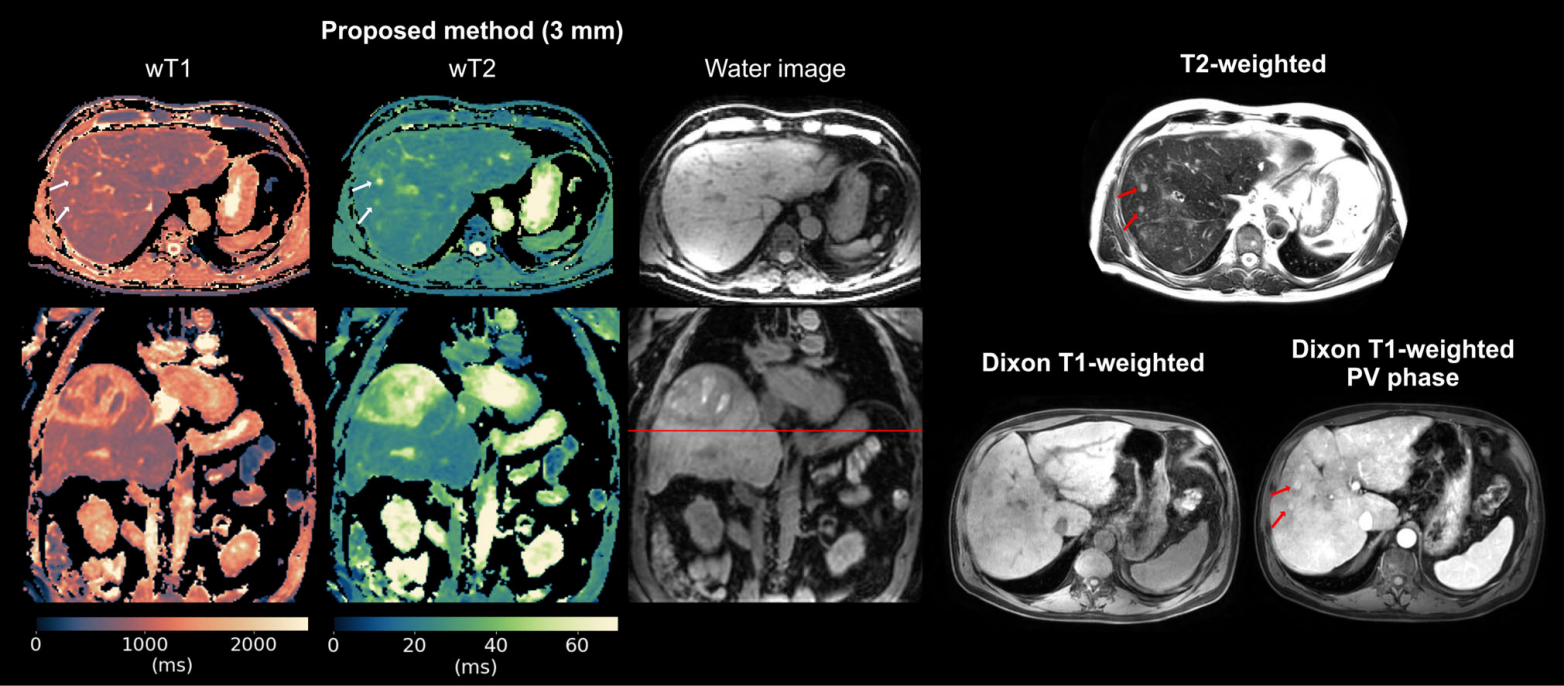
Proposed $wT_{1}$ and $wT_{2}$ mapping in a patient with hepatocellular carcinoma (HCC) following selective internal radiotherapy (SIRT), demonstrating diffuse post‐radiogenic parenchymal changes. 3 mm $wT_{1}$ and $wT_{2}$ maps, as well as PD‐like water images, are shown in axial and coronal views. As a reference from the clinical protocol, $T_{2}$‐weighted, native Dixon $T_{1}$‐weighted, and Dixon $T_{1}$‐weighted images from the portal venous (PV) phase are included. Arrows indicate two small lesions (<1 cm in diameter) visible in the clinical $T_{2}$‐weighted and contrast‐enhanced $T_{1}$‐weighted images. The lesions are well depicted in the proposed $wT_{1}$ and $wT_{2}$ maps. The clinical native Dixon $T_{1}$‐weighted scan required two repetitions due to motion artifacts during the breath‐hold. The slice position is marked by a red line in the coronal view of the water image.

A patient with HCC following selective internal radiotherapy (SIRT) is shown in Figure 9. The proposed method identified two small lesions, each measuring less than 1 cm in diameter, located on a slice inferior to a large tumor and within liver parenchyma exhibiting both post‐radiogenic and cirrhotic changes. The lesions were visible in the $wT_{1}$ and $wT_{2}$ maps and can also be delineated in the contrast‐enhanced Dixon $T_{1}$‐weighted images for reference.

Supplementary Figures S6 and S7 present results from two additional patients, demonstrating the capability of the proposed method to visualize small lesions adjacent to the portal vein and in proximity to the resection margin following partial hepatectomy. Supplementary Figure S8 shows results from a patient with a benign lesion, which was inconclusive on sonography but demonstrated imaging features consistent with a hemangioma on MRI. The $wT_{2}$ map shows high $T_{2}$ values within the lesion, which corresponds to an increase in signal intensity in the clinical $T_{2}$‐weighted image.

## 4 DISCUSSION

The present work proposes a novel method for abdominal simultaneous $wT_{1}$ and $wT_{2}$ mapping during free breathing, with several contributions over previous approaches. First, the method applies a CASPR trajectory enabling flexible isotropic resolution large‐FOV imaging when combined with the Look‐Locker scheme. Second, the Look‐Locker scheme was combined with the mBIR‐4 preparation pulse of varying durations to ensure efficient simultaneous $B_{1}$‐insensitive $T_{1}$ and $T_{2}$ mapping. Finally, a post‐processing pipeline was integrated to facilitate accurate quantification, achieving results comparable to spin echo reference techniques in the phantom.

Previous abdominal free‐breathing methods have predominantly employed the radial stack‐of‐stars trajectory.^30^, ^31^, ^32^, ^33^, ^34^ The radial in‐plane sampling oversamples the k‐space center, reducing motion sensitivity and facilitating motion‐corrected reconstruction. Radial sampling is combined with Cartesian sampling in the third k‐space dimension $k_{z}$, which is typically chosen as the FH direction to align with the primary direction of respiratory motion. Usually, a radial spoke is read out at a fixed angle for all $k_{z}$ profiles in each shot. The approach allows self‐navigation if the contrast does not vary considerably along the acquisition.^31^ The shot duration is therefore determined by the number of $k_{z}$ profiles and depends on the selected acquisition voxel size and FOV in FH direction. The $T_{1}$ relaxation in a Look‐Locker acquisition leads to contrasts variations along the shot, which complicate image reconstruction and can result in image blurring for long shots.^65^, ^66^ Additionally, long shots may complicate fine temporal sampling of the Look‐Locker $T_{1}$ relaxation curve.

In this work, a CASPR approach was chosen over the radial stack‐of‐stars trajectory due to its increased flexibility in pulse sequence design. CASPR is based on Cartesian sampling and samples k‐space profiles in each shot approximately along a spiral.^46^ The FH direction is usually selected as the frequency‐encoding direction in order to achieve similar motion correction properties to the radial stack‐of‐stars trajectory.^37^, ^38^ The shot duration can be set flexible and therefore facilitates a trade‐off between sequence efficiency, motion robustness, and the number of contrasts sampled along the $T_{1}$ relaxation curve. Consequently, a Look‐Locker scheme with CASPR enables isotropic resolution parameter mapping in the abdomen, whereas previous approaches with the radial stack‐of‐stars trajectory have required larger slice thickness and reduced FH coverage.^31^, ^32^, ^34^

Furthermore, CASPR enables acquisition at different matrix sizes using a similar pulse sequence scheme without adapting the duration of each individual shot. The use of the same pulse sequence scheme allows the use of a single dictionary across spatial resolutions or FOV settings, avoiding computationally expensive Bloch simulations. Increasing the matrix size requires only the alternation of the sampled phase encoding locations in the $k_{y}-k_{z}$ grid without the need to change other pulse sequence timings. Therefore, the dictionary computation can be performed only once for a given pulse sequence timing. Furthermore, the use of the same pulse sequence scheme preserves properties such as sensitivity to magnetic field inhomogeneities or system imperfections across spatial resolution or FOV settings. The method demonstrated good accuracy and reproducibility across different spatial resolutions in both phantom and in vivo studies. Slightly larger variations were observed in the comparison of the 3.5 and 3 mm acquisitions, likely due to the reduced spatial resolution and partial volume effects. The ability to select different spatial resolutions and FOV settings without changing the pulse sequence scheme gives the method flexibility when used in a clinical workflow in patients of different sizes or when applied in different anatomies.

The pulse sequence is designed for efficiency and low sensitivity to $B_{1}$ inhomogeneities. The Look‐Locker readout of around 1.4 s is followed by a pause of around 1.4 or 1.6 s for the first or other blocks. The incomplete $T_{1}$ recovery increases efficiency,^67^ while the pause and the small 3° flip angle were designed to mitigate $B_{1}$ sensitivity. Moreover, a dual‐echo bipolar gradient echo readout was employed allowing short $T_{R}$, while correcting for the presence of fat. Two‐echo water‐fat separation for liver water $wT_{1}$ and $wT_{2}$ mapping has recently been proposed in several methods,^8^, ^33^, ^37^, ^68^ which have shown good accuracy in estimating the relaxation properties of the water component, although the methods may not be optimal for accurate fat quantification. An extension of the acquisition to six echoes could enable the additional mapping of proton density fat fraction (PDFF) and $T_{2}^{*}$.

The developed method combines the acquisition scheme with subspace reconstruction, water‐fat separation, and $B_{0}$‐specific dictionary matching. Subspace reconstruction corrects for potential $T_{1}$ relaxation effects during readout and enforces a low‐rank constraint for efficient image reconstruction. Water‐fat separation mitigates confounding effects of fat, while the $B_{0}$‐specific dictionary approach accounts for the residual $B_{0}$ sensitivity of the mBIR‐4 pulse.^33^, ^43^ Experiments showed that $B_{0}$ inhomogeneities primarily impact $wT_{1}$ mapping. The $B_{0}$‐specific dictionary matching effectively corrects for possibly effects within the range of the simulated dictionary.

The proposed 3 mm method at 5:23 min reduces scan time compared to recently proposed free‐breathing simultaneous $wT_{1}$ and $wT_{2}$ methods at 0.55 T^37^ and 3 T^33^ with scan times of 9:25 min and 6:09 min, respectively. At the same time, the method shows a good agreement with Dixon IR‐SE $wT_{1}$ and Dixon SE $wT_{2}$ for a wide range of $T_{1}$ and $T_{2}$ and for different fat fractions in the phantom study. Water‐fat‐separated spin echo reference techniques were only employed in the phantom due to their long scan time. The volunteer study employed MOLLI $T_{1}$ and GRASE $T_{2}$ mapping as vendor‐implemented parameter mapping, which already showed deviations in the phantom study and similar trends in the in vivo study. Both methods are susceptible to confounding effects from fat, but might also be additionally confounded, e.g. by stimulated echoes in GRASE and $T_{2}$ effects in MOLLI.^33^

The proposed method shows similarities to MT methods,^40^ which acquire multiple contrasts and apply low‐rank constraints in reconstruction. However, the proposed approach also differs in key aspects. The proposed method acquires $wT_{1}$ and $wT_{2}$ maps, whereas the presented liver MT method was developed for $wT_{1}$, PDFF, and $R_{2}^{*}$ mapping.^32^ Prior work has explored MT for cardiac simultaneous $T_{1}$ and $T_{2}$ mapping, but only in 2D acquisitions without water‐fat separation.^40^ Liver MT employs a low‐rank tensor model with spatial, respiratory, temporal $T_{1}$, and multi‐echo dimensions,^32^ whereas the proposed method applies a subspace reconstruction with low‐rank constraint only in the joint $T_{1}$ and $T_{2}$ temporal dimension. Liver MT was performed based on a radial stack‐of‐stars trajectory.^32^ A recent method on abdominal MT for radiotherapy treatment planning employed a CASPR trajectory.^38^ The CASPR trajectory used in the MT method acquires 10 profiles per inward spiral interleaf, thereby achieving a higher temporal resolution compared to the 60 profiles per outward spiral interleaf in the proposed method. The higher temporal resolution facilitates motion‐resolved reconstruction, but comes at the cost of lower acquisition efficiency due to increased oversampling. In the MT approach, the oversampled k‐space data enabled the derivation of a subspace basis for reconstruction directly from the acquired data and allowed motion estimation from a real‐time image series. Fundamentally, the MT method for radiotherapy treatment planning and the proposed method target different applications. The MT method was developed to generate motion‐resolved $T_{1}$‐, $T_{2}$‐, and PD‐weighted images. In contrast, the proposed method is designed for motion‐compensated relaxometry. The proposed method employs a dedicated pulse sequence scheme to acquire multiple $T_{1}$ and $T_{2}$ contrasts for accurate parameter mapping and does not reconstruct dynamic motion‐resolved images. The proposed method uses furthermore dictionary matching for parameter mapping similar to MR fingerprinting, allowing direct integration of confounding factors such as $B_{0}$ inhomogeneity, whereas the liver MT method uses an analytical signal model.^32^

The present work includes a patient study with comparable reconstruction quality compared to the volunteer study. The proposed method with 3 mm isotropic resolution effectively visualized small lesions under 1 cm in diameter. Lesions were identified in one HCC patient following SIRT with diffuse post‐radiogenic parenchymal changes, in one patient after partial hepatectomy directly at the resection margin and in another HCC patient near the hepatic hilum and portal vein. The findings highlight the potential of the proposed 3D free‐breathing $wT_{1}$ and $wT_{2}$ mapping in challenging areas due to overlapping structures and motion artifacts in conventional scans. However, further studies with larger patient cohorts are warranted. In addition, the method may be applied in patients with diffuse liver disease enabling an assessment of the whole liver and neighboring organs. The 3.5 mm method with reduced spatial resolution may be sufficient for diffuse liver disease, leading to a possible reduction in scan time to less than 4 min.

The present work has some limitations. First, sequence parameters could be further optimized by adjusting the durations of ${\text{TD}}_{1}$ and ${\text{TD}}_{2}$, or modifying preparation pulse durations to enhance $T_{1}$ and $T_{2}$ sensitivity and $B_{0}$ and $B_{1}$ insensitivity. The sequence parameters were not further optimized in the present work due to the multidimensional parameter space and computationally intensive Bloch simulations. Second, motion navigation was performed based on the respiratory motion signal extracted from external sensors. A respiratory motion‐tracking camera was employed, which tracks motion based on the surface of the subject.^57^ CASPR oversamples the k‐space center, which would also allow self‐navigation, but the varying contrasts of the Look‐Locker acquisition complicate motion estimation. Phase‐based^69^ or model‐based approaches^32^ may be applicable, but may require additional sampled echoes. Third, a soft‐gated image reconstruction was applied to reconstruct parameter maps of the end‐expiration motion state. Although all data were considered and weighted according to the motion signal, there exist more efficient motion‐corrected reconstruction techniques, e.g., techniques that involve reconstruction of all motion states and subsequent non‐rigid registration to a single motion state.^70^ Furthermore, the proposed method includes correction for respiratory motion only. Other sources of motion, such as cardiac or peristaltic motion, were not explicitly addressed and may have contributed to residual stripe artifacts or localized image blurring. Fourth, $wT_{1}$ maps exhibited slightly higher spatial resolution than $wT_{2}$ maps, possibly due to more sampled $T_{1}$ contrasts using the Look‐Locker scheme compared to the four different $T_{2}$ contrasts achieved by changing the duration of the preparation pulse.

## 5 CONCLUSION

A novel method for abdominal simultaneous 3D $wT_{1}$ and $wT_{2}$ mapping during free breathing was developed in this work. CASPR offers flexibility in pulse sequence design, enabling isotropic resolution of 3 mm and large FOV coverage, especially in FH direction, in a scan time of about 5 minutes. By combining the proposed pulse sequence design with subspace reconstruction, water‐fat separation and dictionary matching, the approach has demonstrated high accuracy in phantom and volunteer studies as well as its feasibility in oncological patients with abdominal pathologies.

## CONFLICT OF INTEREST STATEMENT

Kilian Weiss is employee of Philips GmbH Market DACH, Jakob Meineke is employee of Philips Innovative Technologies GmbH, Dimitrios Karampinos received grant support from Philips Healthcare while at the Technical University of Munich.

## Supporting information




Data S1.
**Table S1:** Sequence parameters for the institutional clinical liver protocol. PV: portal venous.
**Table S2**: Mean and range of $wT_{1}$ and $wT_{2}$ times in liver, pancreas, spleen and muscle for n=5 volunteers and the proposed method at 3 mm spatial resolution.
**Table S3**: Clinical scoring on a 5‐point Likert scale for the patient cohort (n=9). Overall image quality was evaluated based on the impact of motion artifacts, susceptibility effects, $B_{1}$ inhomogeneity, and other artifacts. The image sharpness was evaluated based on the perceived versus nominal spatial resolution.
**Figure S1**: Simulated $T_{1}$ and $T_{2}$ estimation error as a function of $B_{1}$ inhomogeneity. Estimation errors are shown across representative abdominal relaxation times. Bloch‐simulated signals were matched to the pre‐computed dictionary with $B_{1}=1$. The differences between estimated and true relaxation times indicate that liver $T_{1}$ and $T_{2}$ mapping is largely robust to typical $B_{1}$ inhomogeneities, but sensitivity increases for longer $T_{1}$ and $T_{2}$ values.
**Figure S2**: Phantom measurements. The differences between the proposed method and the references (Dixon IR‐SE $wT_{1}$ and Dixon SE $wT_{2}$ mapping) are compared with MOLLI $T_{1}$ and GRASE $T_{2}$ as a function of fat fraction. While the differences between the proposed method and the references remain relatively stable across the fat fractions, the difference between MOLLI and GRASE to the respective reference tends to positively correlate with the fat fraction.
**Figure S3**: Phantom measurements. Bland‐Altman analysis of the proposed method at different spatial resolutions shows minimal differences, indicating good reproducibility. (A) Comparison between scans at 2.5 and 3 mm resolution. (B) Comparison between scans at 3.5 and 3 mm resolution.
**Figure S4**: Volunteer measurements. Bland‐Altman analysis to investigate the reproducibility of the proposed method at three different spatial resolutions without repositioning in five representative volunteers. ROIs in the liver, pancreas, spleen, and muscle are compared at (a) 2.5 mm and (b) 3.5 mm isotropic resolution against the 3 mm acquisition. The analysis shows good agreement between the different scans with the highest variance between the scans for the pancreatic ROIs.
**Figure S5**: Coefficient of variation (CV) for ROI measurements at three different spatial resolutions in five volunteers. ROIs were located in the liver, pancreas, spleen and muscle, with two ROIs in the liver (denoted as liver 1 and liver 2). The CV obtained with each technique is indicated by black crosses for the different ROIs and the total CV by the dashed line. $wT_{1}$ showed ${\text{CV}}_{\text{technique}}=1.9\%$ across multiple abdominal organs, volunteers and spatial resolutions, while $wT_{2}$ showed ${\text{CV}}_{\text{technique}}=4.1\%$.
**Figure S6**: Proposed $wT_{1}$ and $wT_{2}$ mapping in a patient with hepatocellular carcinoma located in liver segment 4b, directly adjacent to the liver hilum and the portal vein. Strong $B_{0}$ field inhomogeneities were observed in the lower part of the FOV, likely due to a hip implant. 3 mm $wT_{1}$ and $wT_{2}$ maps, as well as PD‐like water images, are shown in axial and coronal views. As a reference from the clinical protocol, $T_{2}$‐weighted, native Dixon $T_{1}$‐weighted, and Dixon $T_{1}$‐weighted images from the arterial phase are included. Arrows indicate a lesion visible in the $wT_{1}$ and $wT_{2}$ maps, as well as in the $T_{2}$‐weighted and arterial‐phase $T_{1}$‐weighted images of the clinical protocol.
**Figure S7**: Proposed $wT_{1}$ and $wT_{2}$ mapping in a patient with a history of rectal carcinoma and liver metastases following partial hepatectomy. Currently, imaging reveals a small recurrence directly adjacent to the resection margin. 3 mm $wT_{1}$ and $wT_{2}$ maps, as well as PD‐like water images, are presented in axial and coronal views and compared to $T_{2}$‐weighted and native Dixon $T_{1}$‐weighted images from the clinical protocol. Arrows indicate a lesion in the upper liver region.
**Figure S8**: Proposed $wT_{1}$ and $wT_{2}$ mapping in a patient with a suspected liver lesion that was inconclusive on sonography. 3 mm $wT_{1}$ and $wT_{2}$ maps, as well as PD‐like water images, are presented in axial and coronal views and compared to $T_{2}$‐weighted and native Dixon $T_{1}$‐weighted images from the clinical protocol. Arrows indicate the lesion that likely represents a hemangioma. The lesion appears hyperintense on the $T_{2}$‐weighted images and exhibits an increased $wT_{2}$ in the proposed maps.

**Video S1.** MP4 video of axial slices for the proposed $wT_{1}$ and $wT_{2}$ maps as well as the PD‐like water image in a representative subject.

**Video S2.** MP4 video of coronal slices for the proposed $wT_{1}$ and $wT_{2}$ maps as well as the PD‐like water image in a representative subject.

## Data Availability

The source code and example phantom data will be made publicly available: https://github.com/BMRRgroup/abdominal‐t1t2‐mapping. The in vivo data are not publicly available due to privacy or ethical restrictions.
