# Supplementary material for: Abdominal simultaneous 3D water T1 and T2 mapping using a free‐breathing Cartesian acquisition with spiral profile ordering
Source: Magn Reson Med. 2025 Sep 5;95(1):268–85. doi: 10.1002/mrm.70040 (PMC12620181; doi:10.1002/mrm.70040)
Supplement: Supplementary file 1 — Data S1. Table S1: Sequence parameters for the institutional clinical liver protocol. PV: portal venous. Table S2: Mean and range of wT1 and wT2 times in liver, pancreas, spleen and muscle for n=5 volunteers and the proposed method at 3 mm spatial resolution. Table S3: Clinical scoring on a 5‐point Likert scale for the patient cohort (n=9). Overall image quality was evaluated based on the impact of motion artifacts, susceptibility effects, B1 inhomogeneity, and other artifacts. The image sharpness was evaluated based on the perceived versus nominal spatial resolution. Figure S1: Simulated T1 and T2 estimation error as a function of B1 inhomogeneity. Estimation errors are shown across representative abdominal relaxation times. Bloch‐simulated signals were matched to the pre‐computed dictionary with B1=1. The differences between estimated and true relaxation times indicate that liver T1 and T2 mapping is largely robust to typical B1 inhomogeneities, but sensitivity increases for longer T1 and T2 values. Figure S2: Phantom measurements. The differences between the proposed method and the references (Dixon IR‐SE wT1 and Dixon SE wT2 mapping) are compared with MOLLI T1 and GRASE T2 as a function of fat fraction. While the differences between the proposed method and the references remain relatively stable across the fat fractions, the difference between MOLLI and GRASE to the respective reference tends to positively correlate with the fat fraction. Figure S3: Phantom measurements. Bland‐Altman analysis of the proposed method at different spatial resolutions shows minimal differences, indicating good reproducibility. (A) Comparison between scans at 2.5 and 3 mm resolution. (B) Comparison between scans at 3.5 and 3 mm resolution. Figure S4: Volunteer measurements. Bland‐Altman analysis to investigate the reproducibility of the proposed method at three different spatial resolutions without repositioning in five representative volunteers. ROIs in the liver, pancreas, spleen, [file MRM-95-268-s003.pdf]

Abdominal simultaneous 3D water T1 and T2 mapping using a free-breathing Cartesian acquisition with spiral profile ordering

Supplementary information

	T_2-weighted	T_1-weighted Native & PV phase	T_1-weighted Arterial phase
Motion compensation	Triggered	Breath-hold	Breath-hold
Voxel size (mm^3)	1.3×1.3×4 (2D)	1.5×1.6×5 (3D)	1.7×2.2×5 (3D)
FOV (mm^3)	400×400×264	400×318×265	400×319×265
TE (ms)	126	1.3/2.4	1.3/2.3
TR (ms)	2067	3.7	3.5
FA (°)	90	10	12
Acceleration	SENSE (R=2.5)	CS-SENSE (R=6)	CS-SENSE (R=6)
Scan time (min:s)	3:36 (nominal)	00:13	00:07

Table S1: Sequence parameters for the institutional clinical liver protocol. PV: portal venous

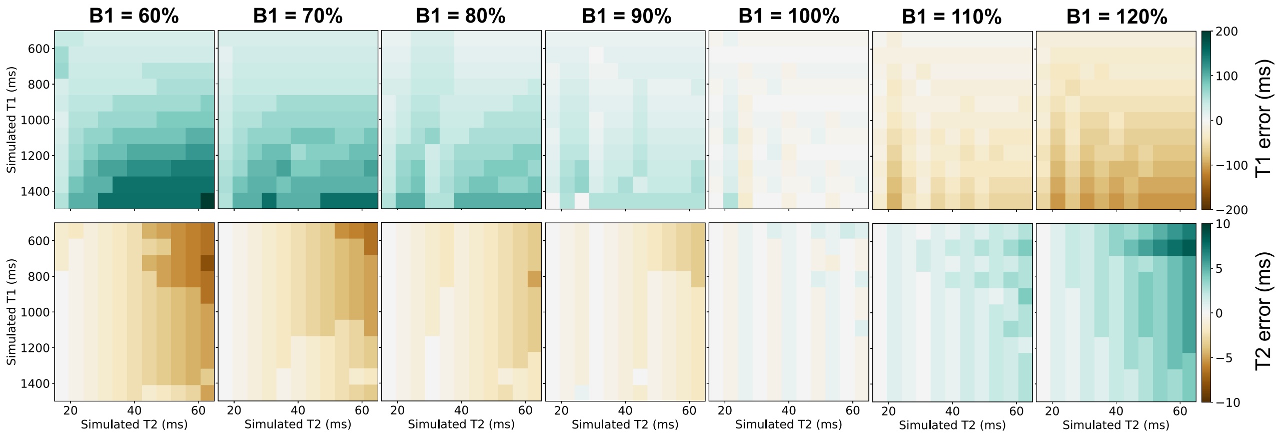

Figure S1: Simulated T_1 and T_2 estimation error as a function of B_1 inhomogeneity. Estimation errors are shown across representative abdominal relaxation times. Bloch-simulated signals were matched to the pre-computed dictionary with $B_1 = 1$. The differences between estimated and true relaxation times indicate that liver T_1 and T_2 mapping is largely robust to typical B_1 inhomogeneities, but sensitivity increases for longer T_1 and T_2 values.

	wT_1 (ms)	wT_2 (ms)
Liver	910 (789-1016)	28.2 (20.9-32.7)
Pancreas	892 (860-956)	42.5 (36.9-47.5)
Spleen	1418 (1295-1475)	51.3 (37.7-56.0)
Muscle	1284 (1262-1312)	29.0 (27.6-29.8)

Table S2: Mean and range of wT_1 and wT_2 times in liver, pancreas, spleen and muscle for $n = 5$ volunteers and the proposed method at 3 mm spatial resolution.

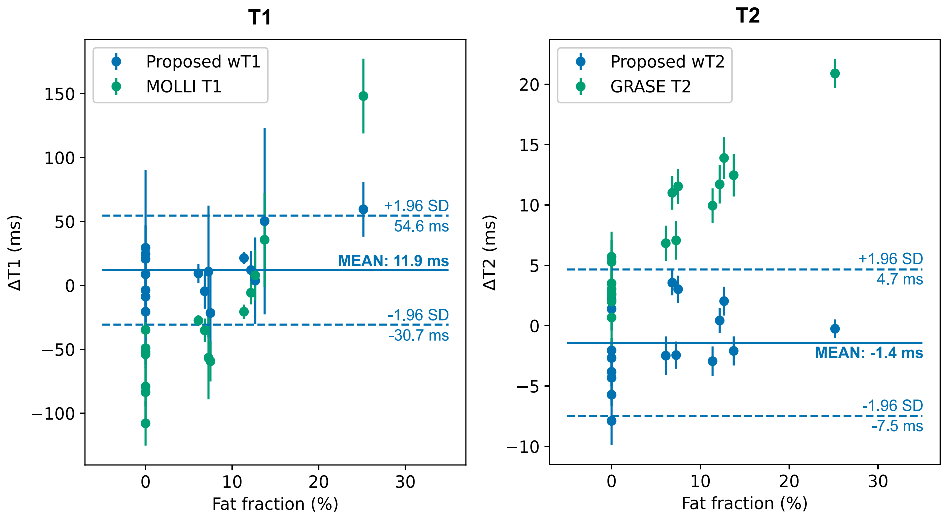

Figure S2: Phantom measurements. The differences between the proposed method and the references (Dixon IR-SE wT_1 and Dixon SE wT_2 mapping) are compared with MOLLI T_1 and GRASE T_2 as a function of fat fraction. While the differences between the proposed method and the references remain relatively stable across the fat fractions, the difference between MOLLI and GRASE to the respective reference tends to positively correlate with the fat fraction.

	**Overall image quality**	**Image sharpness**
1 (non-diagnostic)	-	-
2 (poor)	-	-
3 (moderate)	1	1
4 (good)	3	2
5 (excellent)	5	6

Table S3: Clinical scoring on a 5-point Likert scale for the patient cohort ($n = 9$). Overall image quality was evaluated based on the impact of motion artifacts, susceptibility effects, B_1 inhomogeneity, and other artifacts. The image sharpness was evaluated based on the perceived versus nominal spatial resolution.

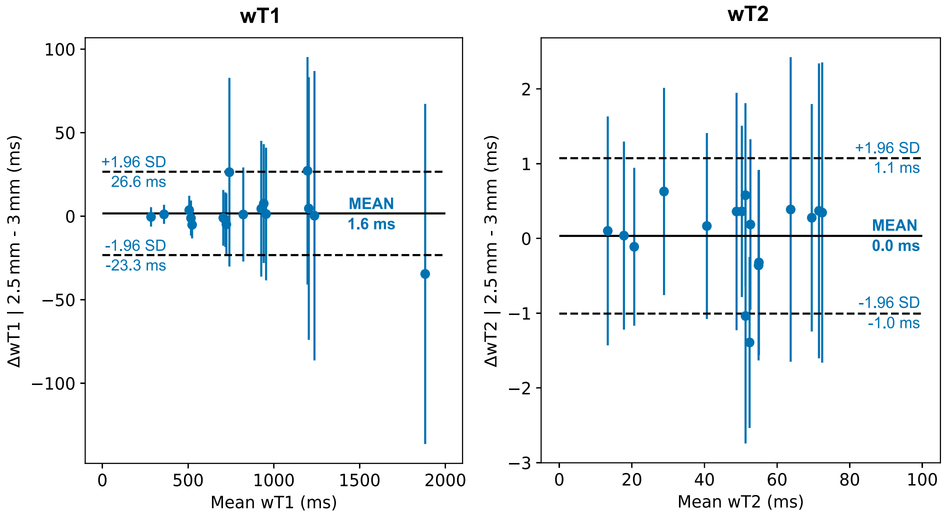

(a) Bland-Altman analysis for 2.5 mm and 3 mm acquisitions

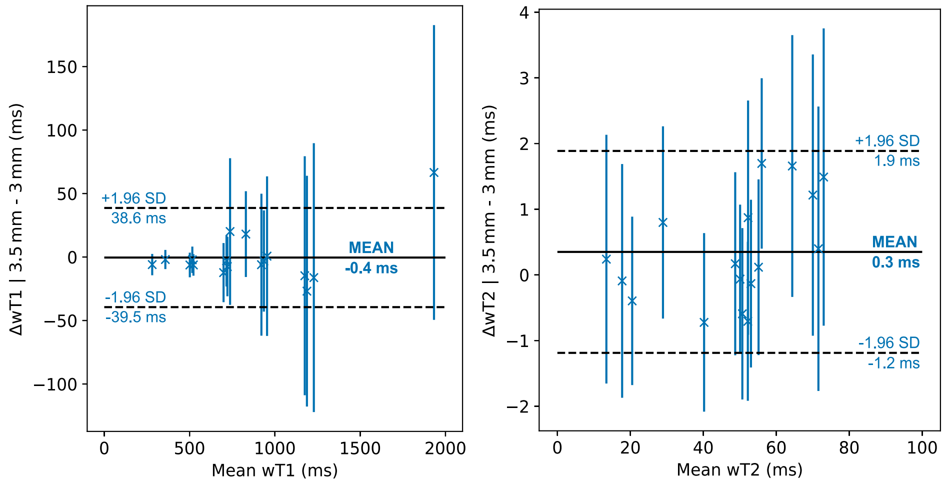

(b) Bland-Altman analysis for 3.5 mm and 3 mm acquisitions

Figure S3: Phantom measurements. Bland-Altman analysis of the proposed method at different spatial resolutions shows minimal differences, indicating good reproducibility. (A) Comparison between scans at 2.5 mm and 3 mm resolution. (B) Comparison between scans at 3.5 mm and 3 mm resolution.

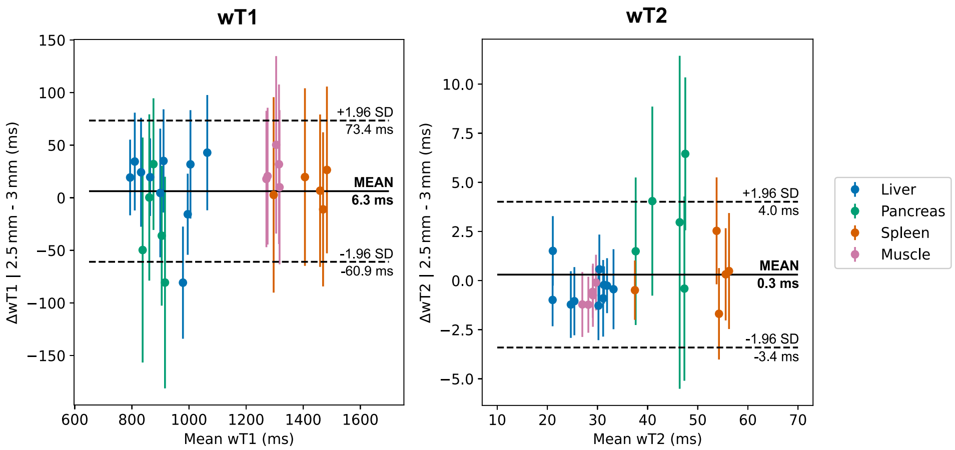

(a) Bland-Altman analysis for 2.5 mm and 3 mm acquisitions

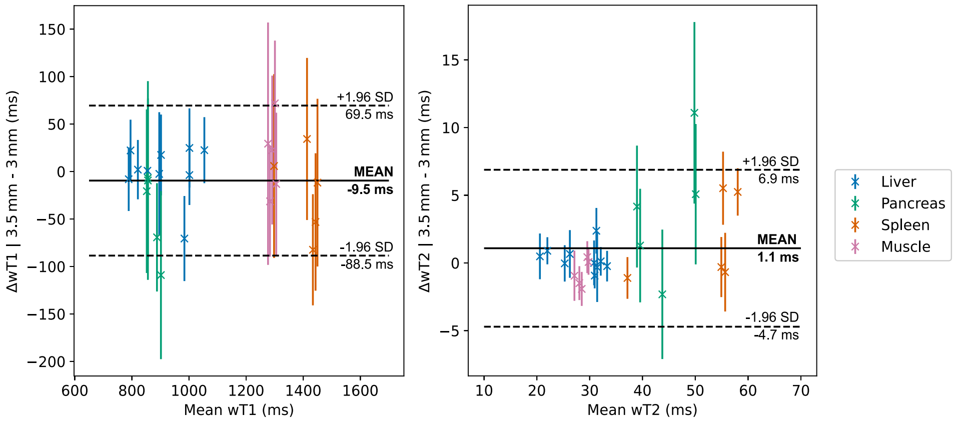

(b) Bland-Altman analysis for 3.5 mm and 3 mm acquisitions

Figure S4: Volunteer measurements. Bland-Altman analysis to investigate the reproducibility of the proposed method at three different spatial resolutions without repositioning in five representative volunteers. ROIs in the liver, pancreas, spleen, and muscle are compared at (a) 2.5 mm and (b) 3.5 mm isotropic resolution against the 3 mm acquisition. The analysis shows good agreement between the different scans with the highest variance between the scans for the pancreatic ROIs.

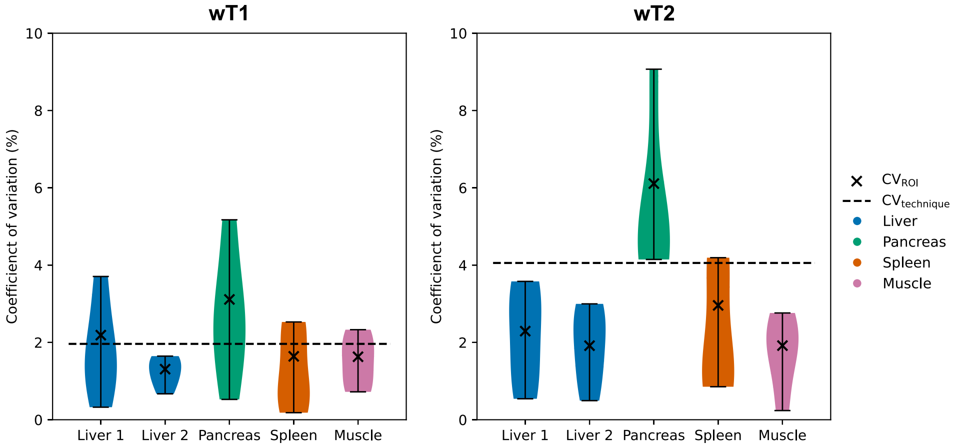

Figure S5: Coefficient of variation (CV) for ROI measurements at three different spatial resolutions in five volunteers. ROIs were located in the liver, pancreas, spleen and muscle, with two ROIs in the liver (denoted as liver 1 and liver 2). The CV obtained with each technique is indicated by black crosses for the different ROIs and the total CV by the dashed line. wT_1 showed $\mathrm{CV}_{\mathrm{technique}} = 1.9\%$ across multiple abdominal organs, volunteers and spatial resolutions, while wT_2 showed $\mathrm{CV}_{\mathrm{technique}} = 4.1\%$.

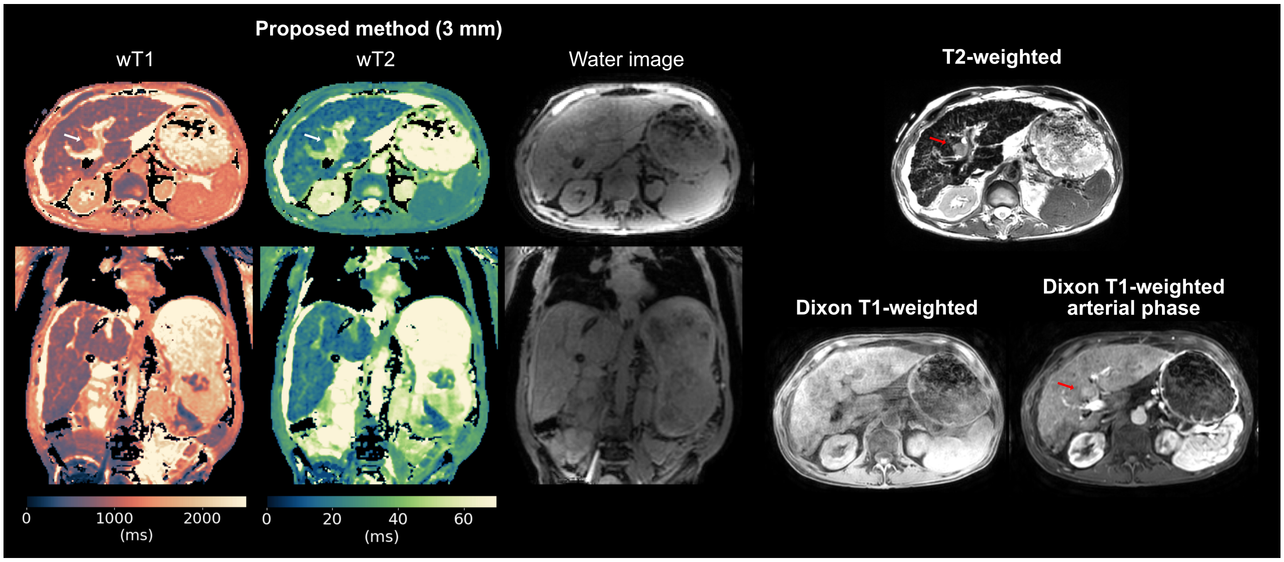

Figure S6: Proposed wT_1 and wT_2 mapping in a patient with hepatocellular carcinoma located in liver segment 4b, directly adjacent to the liver hilum and the portal vein. Strong B_0 field inhomogeneities were observed in the lower part of the FOV, likely due to a hip implant. 3 mm wT_1 and wT_2 maps, as well as PD-like water images, are shown in axial and coronal views. As a reference from the clinical protocol, T_2-weighted, native Dixon T_1-weighted, and Dixon T_1-weighted images from the arterial phase are included. Arrows indicate a lesion visible in the wT_1 and wT_2 maps, as well as in the T_2-weighted and arterial-phase T_1-weighted images of the clinical protocol.

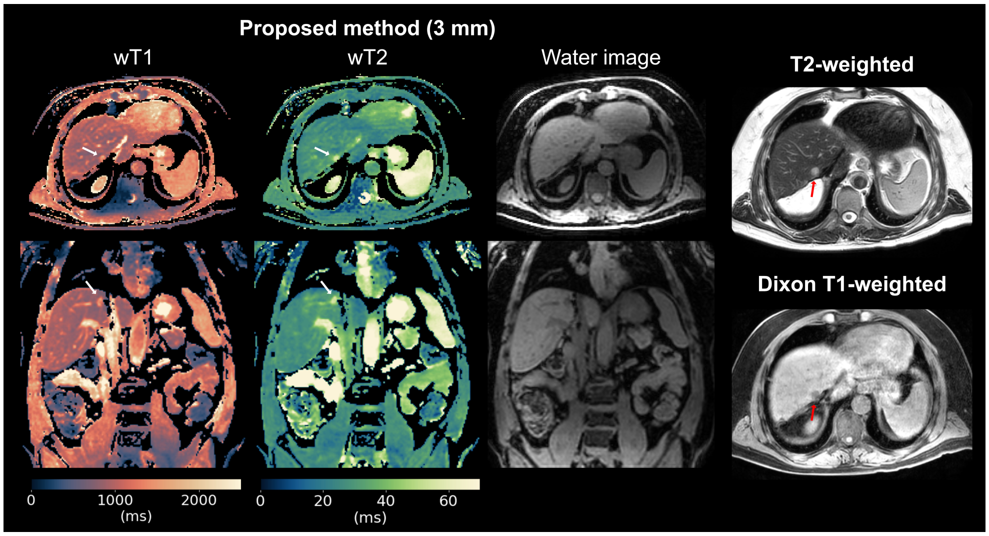

Figure S7: Proposed wT_1 and wT_2 mapping in a patient with a history of rectal carcinoma and liver metastases following partial hepatectomy. Currently, imaging reveals a small recurrence directly adjacent to the resection margin. 3 mm wT_1 and wT_2 maps, as well as PD-like water images, are presented in axial and coronal views and compared to T_2-weighted and native Dixon T_1-weighted images from the clinical protocol. Arrows indicate a lesion in the upper liver region.

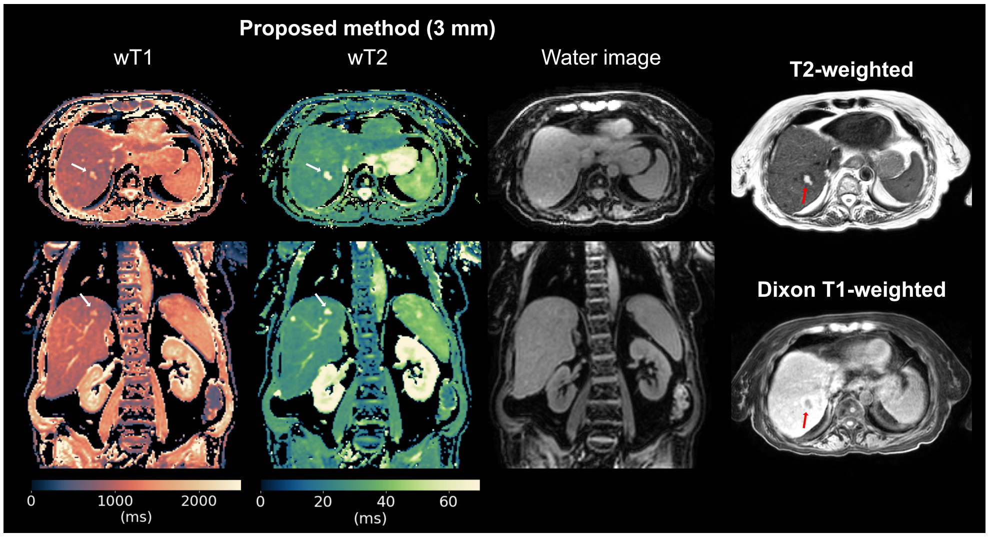

Figure S8: Proposed wT_1 and wT_2 mapping in a patient with a suspected liver lesion that was inconclusive on sonography. 3 mm wT_1 and wT_2 maps, as well as PD-like water images, are presented in axial and coronal views and compared to T_2-weighted and native Dixon T_1-weighted images from the clinical protocol. Arrows indicate the lesion that likely represents a hemangioma. The lesion appears hyperintense on the T_2-weighted images and exhibits an increased wT_2 in the proposed maps.

Figure S9: MP4 video of axial slices for the proposed wT_1 and wT_2 maps as well as the PD-like water image in a representative subject.

Figure S10: MP4 video of coronal slices for the proposed wT_1 and wT_2 maps as well as the PD-like water image in a representative subject.
